# Advances in the Study of Anticipatory Postural Adjustments

**DOI:** 10.3390/brainsci14121219

**Published:** 2024-11-30

**Authors:** William P. Berg

**Affiliations:** Department of Kinesiology, Nutrition and Health, Miami University, 205G Phillips Hall, 420 S. Oak St., Oxford, OH 45056, USA; bergwp@miamioh.edu

## 1. Introduction

Postural stability is critical to the execution of almost any voluntary movement. In fact, “posture is the foundation upon which movement rides” [1]. Posture is controlled by the neuromuscular system in collaboration with visual, vestibular, and somatosensory inputs. One of the ways in which the central nervous system (CNS) minimizes deflections of the body from a desirable alignment is through the use of anticipatory postural adjustments (APAs) [2]. APAs are unconscious preemptive measures taken by the CNS to counteract expected postural perturbations and are critical to functional behavior. For example, when reaching for an object on a shelf, muscles in the trunk and legs activate in advance of muscle activity and movement in the shoulder and arm [3]. APAs occur in both self- and externally-induced perturbations to posture and stability.

APAs were first described in 1967 when Belen’kii et al. [4] reported the existence of electrical activity in the sacrolumbar muscles of the trunk and upper leg prior to rapid shoulder flexion in standing [5]. In recent decades, a considerable amount of research has been conducted in an effort to reveal APA mechanisms and their functional importance. For example, research has demonstrated that APAs are tailored to specific characteristics of focal movements, such as direction [3], load [6], acceleration [7], and velocity [8]. We know that APAs are influenced by conditions such as Parkinson’s disease [9], stroke [10], low back injury [11], and muscle fatigue [12]. Research has also shown that APAs may be compromised in older adults [13], which is perhaps related to the increased fall risk in this population [14]. This is just a small sampling of the work conducted on APAs. 

The purpose of this Special Issue was to provide an avenue for sharing the most recent advances in our understanding of APAs. While the Special Issue includes just four articles and one review paper, it admirably reflects the diversity of current research addressing the role of APAs in postural control. I encourage you to explore the contributions listed below, each of which I briefly describe in the following paragraphs.

1. Mochizuki, L.; Pennone, J.; Bigongiari, A.; Cosme, R.G; Massa, M.; Nicolai Ré, A.H.; Alcântaro, R.P., Jr.; Amadio, A.C. Standing on Elevated Platform Changes Postural Reactive Responses during Arm Movement. *Brain Sci.* **2024**, *14*, 1004. https://doi.org/10.3390/brainsci14101004.

2. Silva-Batista, C.; Lira, J.; Coelho, D.B.; de Lima-Pardini, A.C.; Nucci, M.P.; Mattos, E.C.T.; Magalhaes, F.H.; Barbosa, E.R.; Teixeira, L.A.; Amaro, E., Jr.; Ugrinowitsch, C.; Horak, F.B. Mesencephalic Locomotor Region and Presynaptic Inhibition during Anticipatory Postural Adjustments in People with Parkinson’s Disease. *Brain Sci.* **2024**, *14*, 178. https://doi.org/10.3390/brainsci14020178.

3. Kunimura, H.; Oda, H.; Kawasaki, T.; Tsujinaka, R.; Hamada, N.; Fukuda, S.; Matsuoka, M.; Hiraoka, K. Effect of Laterally Moving Tactile Stimuli to Sole on Anticipatory Postural Adjustment of Gait Initiation in Healthy Males. *Brain Sci.* **2023**, *13*, 1411. https://doi.org/10.3390/brainsci13101411.

4. Marchese, S.M.; Esposti, R.; Farinelli, V.; Ciaccio, C.; De Laurentiis, A.; D’Arrigo, S.; Cavallari, P. Pediatric Slow-Progressive, but Not Non-Progressive Cerebellar Ataxia Delays Intra-Limb Anticipatory Postural Adjustments in the Upper Arm. *Brain Sci.* **2023**, *13*, 620. https://doi.org/10.3390/brainsci13040620.

5. Yousefi, M.; Zivari, S.; Yiou E.; Caderby, T. Effect of Chronic Ankle Instability on the Biomechanical Organization of Gait Initiation: A Systematic Review. *Brain Sci.* **2023**, *13*, 1596. https://doi.org/10.3390/brainsci13111596.

The authors of contribution 1 investigated the conduct of postural adjustments throughout an entire motor action: from the preparatory phase (anticipatory postural adjustment—APA) and focal movement phase (online postural adjustment—OPA) to the compensatory phase (compensatory postural adjustment—CPA) while raising the arms from a standing position. The study is unique in that the authors revised the standard definition of CPAs from those that occur in response to a perturbation to those that occur after the focal movement is completed, thus creating a period they refer to as the OPA. Their goal was to analyze the effects of reduced sensory information and different standing heights on postural muscle activity during the three aforementioned phases. The results indicated that the pattern of postural muscle activation varied across the arm-raising action. Postural muscle activity was the greatest during the OPA phase and lowest during the APA phase. When subjects performed the action from an elevated platform, postural muscles increased activation during APA. The authors concluded that postural control adapts to sensory, motor, and cognitive conditions. Precisely, the increased demand for postural control resulting from performing the task from an elevated support base demands greater flexibility in postural synergies and alters muscle activity.

The authors of the second contribution focused on the symptom of Parkinson’s disease (PD) called freezing of gait (FOG). It is understood that step initiation involves anticipatory postural adjustments (APAs) and that sufferers of FOG (freezers) have a loss of presynaptic inhibition (PSI) during APAs for step initiation. The goal of the study was to identify which locomotor brain region (mesencephalic locomotor region (MLR), supplementary motor area, subthalamic nucleus, and cerebellar locomotor region) explained the loss of PSI. Using an event-related functional magnetic resonance imaging (fMRI) protocol, the authors found that decreased MLR activity during a simulated APA task was related to a higher loss of PSI during APA for step initiation. MLR activity and APA amplitudes during step initiation explained together 45% of the loss of PSI during step initiation in freezers. The authors concluded that deficits in central and spinal inhibitions during APAs may be related to FOG pathophysiology.

The authors of the third contribution to this Special Issue also studied anticipatory postural adjustments (APAs) in gait initiation. The authors examined the effect of laterally moving tactile stimuli (LMTS) administered to the sole of the foot on APAs during gait initiation. It is understood that rhythmic lateral shifting of body weight increases the APA amplitude before gait initiation. The LMTS may mimic the tactile sensation of the sole in rhythmic lateral weight shifting over the feet and thus may interact with postural control processes. Participants took three steps at their preferred pace after a start cue, and the LMTS were delivered to the sole after the cue. The location of the tactile stimuli moved from the left- to the right-most side of the sole and then moved from the right- to the left-most side in a 960 ms cycle, with 16 cycles occurring per trial. The LMTS to the sole decreased the APA amplitude before gait initiation and also made gait initiation slower. The authors contend that the LMTS to the sole slows down gait initiation due to masking of the natural tactile sensation of the sole of the foot and that this reduces the amplitude of the APA. 

The authors of the fourth contribution focused on postural control in children who have ataxia. The authors had recently shown in a previous study that children with genetic slow-progressive ataxia (SlowP) exhibit worse postural control than healthy children. Furthermore and in contrast, the postural control in children with genetic non-progressive ataxia (NonP) resembled that of healthy children. The present study consisted of an effort to extend these findings from inter-limb anticipatory postural adjustments (APAs) to intra-limb APAs. Inter-limb APAs occur, for example, when one reaches with the arm and an APA occurs in the leg. Intra-limb APAs occur in the same limb performing the focal movement. As expected, the authors found that SlowP children exhibited delayed APAs compared to NonP and healthy children. The authors conclude that it is possible that the progressivity of cerebellar disease may be a key factor in determining the impairment in postural control encompassing both inter- and intra-limb APAs. That is, inter- and intra-limb postures presumably share the same control mechanisms.

The fifth contribution to this Special Issue constitutes the only review paper to be included. However, like Contributions 2 and 3, the authors focused on gait initiation. The systematic review was performed to summarize the effects of chronic ankle instability (CAI) on the neuromuscular organization of gait initiation. From 878 articles identified in an initial search, only six were ultimately found that met the inclusion criteria. The findings from these studies indicate that CAI indeed influences gait initiation. Individuals with CAI were distinguished by reaction times, spatiotemporal parameters of anticipatory postural adjustments (APAs) and step execution, ankle–foot kinematics, and muscle activation that differed from healthy control subjects. Especially relevant to this Special Issue, the differences in APA patterns associated with gait initiation argue for the presence of supraspinal motor control alterations in individuals with CAI. This would suggest that CAI should be treated as a global condition (rather than solely as a local musculoskeletal condition) affecting all levels of neuromuscular control. The authors conclude by indicating that rehabilitation programs designed, for instance, to reduce the risk of ankle sprains in individuals with CAI should perhaps focus on restoring normal muscle activation patterns.

## 2. Conclusions

Three of the five articles in this Special Issue address APAs in the context of disease or injury. This will likely remain a research focus in the future as we attempt to fully ascertain the cause and treatment of specific health conditions related to postural control. 

It was noteworthy that all five articles in this Special Issue focused on APAs occurring during self-induced perturbations to posture and stability. Absent were any articles on APAs in externally induced perturbations to posture and stability, such as those that might occur when catching an object or when being bumped into on a crowded sidewalk. Indeed, APAs in externally induced perturbations appear to be less well studied and thus represent a potentially fruitful area of research. Other potentially productive avenues of research include:

- The trainability/adaptability of APAs
- How APAs respond to unpredictable perturbation loads
- New methods for collecting and analyzing APA information (e.g., wearable devices)
- Longitudinal studies to learn if changes in APAs might be predictive of falls or disease
- The pharmacological effects on APAs
- The influence of prostheses usage on APAs
